# The Dual Function of the Fungal Toxin Candidalysin during *Candida albicans*—Macrophage Interaction and Virulence

**DOI:** 10.3390/toxins12080469

**Published:** 2020-07-24

**Authors:** Annika König, Bernhard Hube, Lydia Kasper

**Affiliations:** 1 Department of Microbial Pathogenicity Mechanisms, Leibniz Institute for Natural Product Research and Infection Biology–Hans Knoell Institute, 07745 Jena, Germany; annika.koenig@leibniz-hki.de; 2 Center for Sepsis Control and Care, University Hospital Jena, 07747 Jena, Germany; 3 Institute of Microbiology, Friedrich Schiller University, 07743 Jena, Germany

**Keywords:** candidalysin, cytolytic toxin, dual function, inflammasome activation

## Abstract

The dimorphic fungus *Candida albicans* is both a harmless commensal organism on mucosal surfaces and an opportunistic pathogen. Under certain predisposing conditions, the fungus can overgrow the mucosal microbiome and cause both superficial and life-threatening systemic infections after gaining access to the bloodstream. As the first line of defense of the innate immune response, infecting *C. albicans* cells face macrophages, which mediate the clearance of invading fungi by intracellular killing. However, the fungus has evolved sophisticated strategies to counteract macrophage antimicrobial activities and thus evade immune surveillance. The cytolytic peptide toxin, candidalysin, contributes to this fungal defense machinery by damaging immune cell membranes, providing an escape route from the hostile phagosome environment. Nevertheless, candidalysin also induces NLRP3 inflammasome activation, leading to an increased host-protective pro-inflammatory response in mononuclear phagocytes. Therefore, candidalysin facilitates immune evasion by acting as a classical virulence factor but also contributes to an antifungal immune response, serving as an avirulence factor. In this review, we discuss the role of candidalysin during *C. albicans* infections, focusing on its implications during *C. albicans*-macrophage interactions.

## 1. Introduction

Of the estimated three to five million fungal species existing worldwide, only a tiny portion (less than 0.01%) cause infections in a human host [1,2]. Still, these few species infect more than one billion people worldwide every year [3,4]. These include primary pathogens that cause disease even in a healthy host, like *Histoplasma capsulatum* or *Paracoccidioides brasiliensis*, and opportunistic fungi like *Aspergillus* and *Candida* spp., which need a susceptible host for disease development [5]. Whereas infections with *Aspergillus* spp. are acquired from the environment, infections with *Candida albicans* mainly originate from endogenous reservoirs like the gut [5,6,7,8,9]. 

Systemic (invasive) fungal infections pose a serious and often underestimated global health threat due to their crude mortality rate and rising antifungal resistance [4]. The top 10 invasive fungal infections kill approximately 1.5 million people annually, which is more than deaths caused by tuberculosis or malaria [4]. Many of these infections are typically acquired nosocomially in susceptible hosts, and *Candida* spp. rank as the fourth most common cause, with *C. albicans* accounting for more than half of all *Candida*-induced bloodstream infections [10,11]. Systemic *C. albicans* infections account for more than 400,000 life-threatening infections per year [4].

Both arms of the immune system, innate and adapted immunity, are required for combating *Candida* infections. As significant contributors to innate immunity, macrophages play an important role in immunity against *C. albicans* infections by mediating phagocytosis, clearance of internalized fungi, and recruitment of neutrophils [12,13,14,15,16,17,18]. However, most pathogenic fungi, including *C. albicans*, have evolved elegant strategies to counteract killing by phagocytes. For *C. albicans*, these include hypha formation and the production of the hypha-associated cytolytic peptide toxin candidalysin [19]. In this review, we discuss the role of candidalysin during infection, focusing on its role in the interaction of *C. albicans* with macrophages.

## 2. The Pathogen *C. albicans* and Innate Immune Defense by Macrophages

*C. albicans* usually resides as a harmless commensal on mucosal surfaces such as the gastrointestinal and urogenital tract and the oral cavity [20,21]. Under predisposing conditions such as local immunosuppression or antibiotic treatment, the fungus can cause superficial infections that are comparably harmless and relatively easy to treat [22,23,24]. However, under certain circumstances like systemic immunosuppression, gastrointestinal surgery, central venous catheters, or prolonged stay in intensive care units, the fungus can breach the epithelial barrier, gain access to the bloodstream, and disseminate, causing systemic candidiasis [25,26]. 

In animal models, neutrophils were identified as key players in controlling disseminated candidiasis, and neutropenia is known to be a major risk factor for systemic candidiasis [27,28,29,30,31]. Besides neutrophils, monocyte-derived immune cells play an essential role in host defense against *C. albicans* infections. Several studies showed that monocytes and monocyte-derived immune cells are indispensable for controlling *C. albicans* infections [13,14,15]. Tissue-resident macrophages are needed for innate immune defense against *C. albicans* infections [12]. Patrolling monocytes migrate into infected tissues and differentiate into macrophages and dendritic cells, with the latter bridging innate and adaptive immunity against *C. albicans* by presenting fungal antigens to naive T-cells in lymph nodes [32,33]. Monocytes and macrophages contribute directly to fungal clearance by internalization and subsequent intracellular killing, but they also mediate neutrophil recruitment [14,16,17,18]. 

## 3. Immune Evasion Mechanisms of *C. albicans*

Macrophages recognize fungal-pathogen-associated molecular patterns (PAMPs) via surface pattern recognition receptors (PRRs) and rapidly phagocytose *C. albicans*. One major *C. albicans* PAMP is its cell wall β-glucan, which is detected via the macrophage PRR dectin-1 [34,35]. Upon phagocytosis of fungal cells, the nascent phagosome matures through fusion steps with lysosomes, ultimately forming the phagolysosome. This cellular compartment represents a hostile environment for the fungus characterized by low pH, few nutrients, and antimicrobial activities (such as oxidative, nitrosative, and proteolytic stress) [36,37].

Despite exposure to the macrophage phagosome’s detrimental environment, a fraction of engulfed fungal cells can survive. *C. albicans* counteracts oxidative and nitrosative stress by suppression of ROS generation [38], and production of detoxifying enzymes like superoxide dismutases [39,40,41,42]. Internalized fungal cells rapidly reprogram their metabolism to adapt to nutrient starvation inside the phagosome; this includes the up-regulation of genes involved in alternative carbon use (glyoxylate cycle and fatty acid beta oxidation), or encoding oligopeptide transporters and amino acid permeases whereas genes associated with protein biosynthesis are down-regulated [41,43,44]. The data indicate that *C. albicans* cells experience metabolic starvation inside the phagosome.

In addition, *C. albicans* evades the acidic phagosomal environment; *Candida*-containing phagosomes change from acidic to neutral pH over time [45]. This phagosome neutralization is likely mediated by fungal activities such as the production of neutralizing metabolites [46] or phagosome damage by extending fungal hyphae, which leads to proton leakage [45].

Hypha formation inside macrophages is connected with phagosome damage and the escape of *C. albicans* from these immune cells and immune cell death. *C. albicans* cells engulfed by macrophages rapidly induce hyphal growth, and hyphae are important for many of the above-described immune evasion mechanisms [45,46,47,48,49,50]. Extending hyphae eventually pierce macrophage membranes, causing macrophage cell death due to physical forces, thus providing an escape route for the fungus from the hostile environment inside the phagocyte [49,51,52]. Recent studies showed that *C. albicans* damages macrophages and escapes by inducing early inflammasome-dependent cell death (pyroptosis) [53,54]. Thus, the induction of macrophage pyroptosis can promote the second pathway of fungal escape in addition to the physical damage caused by extending hyphae [51,55,56]. Escaping hyphae consume glucose in the environment rapidly, which provides a third pathway for *C. albicans*-induced macrophage cell death [57].

In combination, these adaptation mechanisms are thought to promote *C. albicans* survival and even proliferation inside macrophages and ultimately allow escape from these immune cells.

## 4. Candidalysin—A Hypha-Specific Cytolytic Peptide Toxin

The ability to change between its two most important morphologies, yeast and hyphal cells, represents one major virulence trait of *C. albicans* [58]. The hyphal growth program is tightly regulated and induced upon multiple stimuli such as body temperature or contact to host surfaces [59]. During this filamentation process, the fungus expresses virulence factors like the adhesin and invasin Als3, the superoxide dismutase Sod5, or secreted aspartic proteases (Sap4–6) [60,61,62]. Hyphae contribute to immune evasion of the fungus following phagocytosis by macrophages and allow invasive growth on host epithelia [63,64,65]. Both processes cause host cell damage, but the damage-mediating fungal factors remained largely unknown. For decades, hydrolases had been thought to be the significant damaging factors of *C. albicans*. In contrast to many bacteria, no pore-forming toxin-like molecules, peptide toxins, or cellular effector proteins were identified in *C. albicans* or any other human pathogenic fungus. As a “toxic surprise” [66], the *C. albicans* toxin candidalysin was recently discovered as the first peptide toxin identified in any human pathogenic fungus [19]. Candidalysin is encoded by the *C. albicans* gene *ECE1*, one of the most highly expressed genes upon hypha formation. The expression of *ECE1* increases within minutes after the induction of filamentous growth up to 10,000-fold [19,67]. *ECE1* is one of the eight core filamentation genes in *C. albicans* induced in response to a wide range of different filamentation stimuli [68], suggesting an important and strictly morphology (hyphal)-associated role during infection. The gene encodes a polypeptide consisting of at least eight peptides separated by lysine-arginine (KR) motifs [69]. The third peptide, candidalysin, is released from the Ece1preproprotein after sequential proteolytic processing by the Golgi-located subtilisin-like protease, Kex2, and the carboxypeptidase, Kex1 [69,70]. The correct processing of the preproprotein is essential for the release of functional candidalysin and, in turn, epithelial damage in vitro and fungal virulence in a model of oropharyngeal candidiasis [70]. This activation mechanism is shared with several bacterial toxins like diphtheria toxin, anthrax toxin protective antigen, or aerolysin, which are similarly activated by proteolytic processing of a precursor protein by subtilisin-like proteases [71,72,73]. Processed candidalysin is secreted and can be detected in culture supernatants and during growth on epithelial cells [19]. Upon complete processing, the toxin consists of 31 amino acids (SIIGIIMGILGNIPQVIQIIMSIVKAFKGNK) and adopts an α-helical structure. It exhibits an amphipathic nature due to containing an N-terminal hydrophobic region and a C-terminal hydrophilic region. The toxin can intercalate into host epithelial membranes through these features, resulting in membrane permeabilization and cell lysis [19]. 

Genetic analyses underlined the importance of candidalysin for fungal mucosal infection since the deletion of the toxin-encoding sequence from the *ECE1* gene completely abolished *C. albicans*-induced damage to epithelial cells in vitro [19]. The same genetic modification attenuated *C. albicans* virulence in a mouse model of oropharyngeal candidiasis and a zebrafish swim bladder infection model, which are two in vivo models of mucosal infection [19]. Importantly, candidalysin or *ECE1*-deletion strains show no defects in hypha formation, adhesion, or invasion properties [19,67]. The data suggest that production of candidalysin, rather than hypha formation per se or secreted hydrolases, are the main mediators of the host cell damage caused by *C. albicans* [74].

## 5. Candidalysin in *C. albicans*—Macrophage Interactions

The membrane-perturbing action of bacterial toxins is well known to play an essential role during confrontation with macrophages by inducing inflammatory responses and inflammatory host cell death [73,74,75,76,77]. 

As the expression of the candidalysin-encoding *ECE1* gene is strongly induced upon *C. albicans* phagocytosis by macrophages [41,43,78], it is likely that mature candidalysin is produced by macrophage-internalized *C. albicans* cells, and toxin-dependent effects occur. *C. albicans* strains lacking the *ECE1* gene or the candidalysin-encoding sequence caused less host cell damage than wild type strains when confronted with primary macrophages over 24 h, pointing to candidalysin-dependent macrophage damage [78]. Hypha formation inside macrophages, and later piercing of macrophage membranes, was unaffected by deletion of the candidalysin-encoding *ECE1* gene; this suggests that the toxin is not needed for physical membrane damage due to hypha extension [53,57,78]. 

In the first hours of *C. albicans*-macrophage interaction, host cell lysis is mainly exerted via caspase-1-dependent pyroptosis, a regulated cell death pathway depending on the activation of the NOD-like receptor protein 3 (NLRP3) inflammasome [53,54,79,80]. Activation of the NLRP3 inflammasome, resulting in secretion of bioactive IL-1β, is triggered by *C. albicans* in myeloid cells like macrophages, dendritic cells, and neutrophils [81,82,83,84], and the NLRP3 inflammasome is an essential component of the host defense against *C. albicans* [55,56]. 

Canonical NLRP3 inflammasome induction requires a priming and subsequent activating step. Detection of microbial ligands like fungal β-glucans or bacterial LPS by host PRRs like dectin-1 or toll-like receptor (TLR)4 leads to inflammasome priming and to the production of pro-IL-1β and pro-IL-18 [85,86]. The inflammasome is then activated, resulting in caspase-1 cleavage into its active form and processing of pro-IL-1β and pro-IL-18 into the mature, pro-inflammatory, secreted forms. In response to bacterial pathogens, the NLRP3 inflammasome can further be activated non-canonically via direct sensing of intracellular LPS by caspase-4 and caspase-5 in humans and caspase-11 in murine cells [86]. In addition, an alternative activation is possible via TLR4-dependent LPS sensing and caspase-8 activation in human monocytes [87]. In both cases, the final cleavage of pro-IL1β and pro-IL-18 into the mature forms is carried out by caspase-1 [86,87]. Apart from this, serine proteases like elastase, cathepsin G, and proteinase 3 are capable of cleaving pro-IL-1β independently of caspase-1 [88]. In in vitro culture conditions, IL-1β maturation was reported to be dependent on caspase-1. However, using in vivo studies, caspase-1-independent IL-1β processing is the prevailing source of mature IL-1β during the acute phase of infection, which is characterized by strong neutrophil infiltration. The contribution of inflammasome-mediated, caspase-1-dependent pro-IL-1β cleavage increases at later time points, which are rather macrophage/monocyte-dominated [88,89,90,91,92].

*C. albicans* hyphae are a necessary but insufficient trigger of inflammasome activation; this suggests that hypha-associated factors and hyphal activities, which remain largely unknown in detail, contribute to inflammasome activation [56,80,93]. Candidalysin has recently been identified as one major trigger of hypha-dependent NLRP3 inflammasome activation in primary human macrophages and murine dendritic cells [78,94]. A synthetic candidalysin peptide is sufficient to induce secretion of mature IL-1β in human and murine macrophages and murine bone marrow-derived dendritic cells in a strictly caspase-1 and NLRP3-dependent manner [78,94]. Infection of macrophages with candidalysin-deficient *C. albicans* mutants proved that candidalysin crucially contributes to *C. albicans*-dependent IL-1β secretion by murine and human macrophages [78,94,95]. 

Candidalysin does not provide the inflammasome-priming signal; instead, it is one of the fungal factors triggering the inflammasome-activating step [78]. The activation of the NLRP3 inflammasome by candidalysin is mediated via potassium efflux, putatively, through toxin-induced membrane perturbances or lesion formation [78]. Potassium efflux is a common inflammasome-activating trigger that is also induced by bacterial toxins [96,97]; this indicates that fungal and bacterial membrane-disturbing toxins can activate similar pro-inflammatory response mechanisms in phagocytes [78,97]. Candidalysin-induced inflammasome activation was not only inhibited by treatment with the potassium channel inhibitor glibenclamide or by the addition of high extracellular potassium [78], but also by the addition of the inflammasome inhibitor MCC950, which directly interacts with the NLRP3 inflammasome by blocking ATP hydrolysis. This suggests that additional mechanisms other than potassium efflux are involved in the candidalysin-mediated inflammasome activation [95].

Despite inducing caspase-1-dependent inflammasome activation, candidalysin does not seem to be a major trigger of caspase-1-dependent pyroptosis in mononuclear cells [78]. Toxin-induced macrophage damage was not reduced in the presence of caspase-1 inhibitors or phagocytes isolated from caspase-1 or NLRP3 knockout mice, and a candidalysin-deficient mutant was still able to induce caspase-1-dependent damage. The data suggest that NLRP3 inflammasome activation is not necessarily coupled to pyroptosis [78]. This is in contrast to many bacterial pore-forming toxins like α-hemolysin from *Staphylococcus aureus* or listeriolysin from *Listeria monocytogenes*, which activate the inflammasome and induce pyroptosis in human and murine monocytic and monocyte-derived cells [97,98,99]. 

The data collected suggest that candidalysin triggers a separate pathway of *C. albicans*-induced host cell damage, likely by directly perturbing host cell membranes but independent of hypha-mediated mechanical host cell rupture and pyroptosis. *C. albicans* dependent pyroptosis instead seems to depend on other factors like fungal cell wall components and fungal morphology [79,100]. In addition, secreted aspartic proteases are known as inflammasome inducers [101] and could potentially contribute to this inflammatory cell death. 

**Figure 1:**
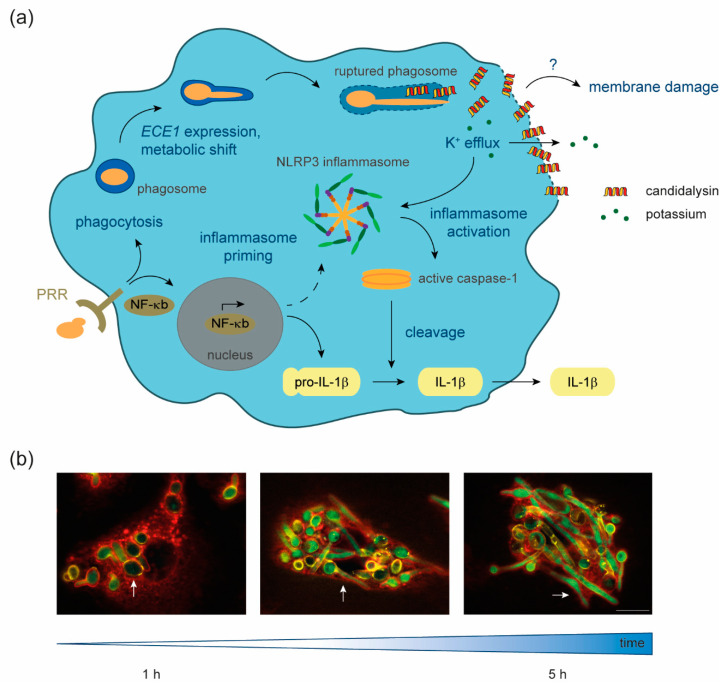
Candidalysin–macrophage interaction. (**a**) Upon recognition of fungal-pathogen-associated molecular patterns (PAMPs) by host pattern recognition receptors (PRR), *Candida albicans* is phagocytosed and NF-κB signaling is induced. NF-κB signal transduction leads to the formation of the primed NLRP3 inflammasome and the production of pro-IL-1β. Inside the phagosome, *C. albicans* cells undergo a metabolic shift to adapt to nutrient limitation, form hyphae, express *ECE1*, and produce the polypeptide Ece1, which is further processed into candidalysin. The filamentation of the fungus inside the phagosome leads to phagosomal membrane damage and eventually hyphal outgrowth. Our data suggest that candidalysin accumulates in phagocyte membranes, facilitating ion fluxes such as potassium efflux, which in turn activates the primed inflammasome. This activation leads to cleavage of pro-caspase-1 into the enzymatically active form, which processes pro-IL-1β into the mature pro-inflammatory IL-1β, which is then secreted. What remains unknown is the exact mechanism through which candidalysin causes membrane damage. (**b**) Fluorescence microscopy images of *C. albicans* cells, which express GFP under control of the *ECE1* promoter, internalized by primary human monocyte-derived macrophages. Over 1 to 5 h, ingested yeast cells (white arrows) start to filament and induce *ECE1* transcription (green). Green, GFP; red, Concanavalin A lectin staining of host cells; yellow, Calcofluor white fungal cell wall staining. The white scale bar represents 10 µm and applies to all fluorescence microscopy images.

In summary, the currently available data shed light on candidalysin functions in macrophage membrane damage and inflammasome activation (Figure 1). Many aspects of the toxin action in these immune cells remain to be elucidated. Candidalysin will likely be secreted by growing hyphae inside the phagosome [19], but the subcellular localization inside macrophages and the mechanisms of toxin distribution within the host cell are unknown. As the toxin seems to be mostly dispensable for damage of the phagosomal membrane by growing hyphae [45], it seems that the phagosomal membrane is not the main target of this fungal toxin.

## 6. Dual Function of Candidalysin during Infection

By taking the candidalysin–macrophage interaction as an example, candidalysin can be seen as a microbial factor that exhibits a dual function during interaction with the host. It provides a mechanism for host cell lysis, contributing to escape from these immune cells. Through activation of the NLRP3 inflammasome, it provokes a pro-inflammatory host-protective response that can be beneficial for fungal clearance (Figure 1) [78]. This combination of effects that are both beneficial and detrimental to the host has also been proposed for the action of bacterial pore-forming toxins during the interaction with macrophages [76]. 

Mouse infection experiments with *C. albicans* mutants lacking *ECE1*, or the candidalysin-encoding sequence only, showed that the candidalysin-dependent induction of IL-1β release transfers from the macrophage in vitro infection model to in vivo models of systemic candidiasis. These experiments showed that candidalysin is required for host IL-1β release in murine kidneys and neutrophil recruitment [30,78]. Similarly, candidalysin-induced IL-1β production by brain microglia can induce antifungal immunity by promoting neutrophil recruitment [18]. 

The dual function model of candidalysin can also be transferred to the interaction of *C. albicans* with epithelial and endothelial barriers. In a mouse model of oral infections, the toxin showed to be critically important for damage induction and the establishment of infection [19]. Candidalysin-dependent damage has been seen in in vitro models of oral, vaginal, and intestinal epithelial, as well as endothelial models [19,30,65,102]. These data represent the toxin’s function as a classical virulence factor [19,103]. However, candidalysin simultaneously activates epithelial PI3K/Akt, NF-κB, p38, JNK, and ERK1/2 MAPK signaling cascades. It thereby elicits a pro-inflammatory response that contributes to the recruitment of immune cells like macrophages, Th17 cells, and neutrophils to the site of infections. It mediates a protective crosstalk via the latter [19,104,105,106,107]. This danger response is also activated by candidalysin in endothelial cells and vaginal cells [30,102]. Potentially, this is mostly mediated via the release of alarmins and antimicrobial peptides in epithelial cells [108]. 

**Figure 2:**
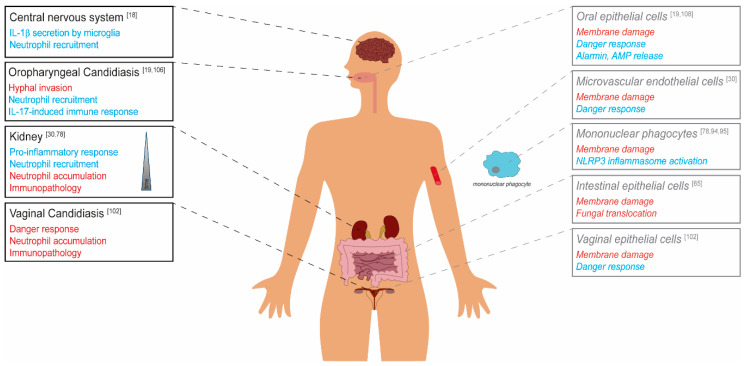
The role of candidalysin in *C. albicans* virulence. Candidalysin-dependent effects on the *C. albicans* virulence potential are depicted in red (detrimental for the host, classical virulence factor) and blue (beneficial for the host, avirulence factor) for the respective body site/organ. Black and strong colors show in vivo data derived from murine models. Grey, light colors, and typesetting in italics represent in vitro data from cell culture studies.

Candidalysin can be seen both as a virulence factor that helps to evade innate immune responses or to breach host barriers, but also as an avirulence factor that can activate host-protective responses in the immunocompetent host and thus limit the pathogen’s virulence (Figure 2) [109,110].

Depending on the infection site, candidalysin exhibits differential effects on fungal virulence. The toxin contributes to damage of host cell membranes, which is connected with fungal invasion, translocation through barriers and escape from phagocytes. The activation of host responses like the epithelial danger response pathway or the phagocyte NLRP3 inflammasome, and resulting neutrophil recruitment, in many cases leads to a protective host response. In contrast, massive neutrophil infiltration during vaginal infection and in later stages of systemic infection can cause immunopathology.

Under certain circumstances, the immune system’s activation can also promote fungal virulence and worsen the infection outcome. During disseminated candidiasis, for example, neutrophil recruitment stimulated by candidalysin increases mouse mortality during later infection stages, which is likely related to immunopathological effects [30]. Similarly, a strong candidalysin-mediated infiltration of neutrophils is responsible for the typical immunopathology of vaginal *C. albicans* infections [102].

As discussed above, candidalysin expression is associated with filamentation of the fungus [19,67,68]; however, filaments are not the dominating phenotype in all host niches infected by *C. albicans*. For example, hyphae dominate in brain and kidney tissue but seem to be absent in the liver and spleen during disseminated candidiasis in a mouse model of systemic infection [111]. The murine gut is predominantly colonized by yeast cells or a mixture of yeast and hyphae [112,113]. In addition, the inhibition of filamentation by external cues or genetic modification will reduce candidalysin production [114,115]. Thus, candidalysin-induced effects will likely not only depend on niche-specific host responses but also niche-specific levels of filamentation and expression of candidalysin in the respective infection environment.

## 7. Conclusions

Macrophages are, besides neutrophils, crucial for combating disseminated candidiasis. Fungal killing is mediated by a combination of antimicrobial activities within the phagosome. The fungus can counteract these attempts by producing hyphae, which induce pyroptosis, mechanically stretch, and ultimately lyse the phagosomal membrane, thereby inducing immune cell death, further supported by fungal glucose consumption. 

The cytolytic peptide toxin candidalysin contributes to macrophage lysis but also mediates the induction of pro-inflammatory cytokine release via the NLRP3 inflammasome. This, as well as its diverse implications during oral, vaginal, and systemic infections, highlights the dual function of this toxin as a classical virulence factor and an avirulence factor during the *C. albicans*-macrophage interaction, mucosal, and systemic infection.

## References

[1] Kohler J.R., Hube B., Puccia R., Casadevall A., Perfect J.R. (2017). Fungi that Infect Humans. Microbiol. Spectr..

[2] Blackwell M. (2011). The fungi: 1, 2, 3 … 5.1 million species?. Am. J. Bot..

[3] Havlickova B., Czaika V.A., Friedrich M. (2008). Epidemiological trends in skin mycoses worldwide. Mycoses.

[4] Brown G.D., Denning D.W., Gow N.A., Levitz S.M., Netea M.G., White T.C. (2012). Hidden killers: Human fungal infections. Sci. Transl. Med..

[5] Kohler J.R., Casadevall A., Perfect J. (2014). The spectrum of fungi that infects humans. Cold Spring Harb. Perspect. Med..

[6] Nucci M., Anaissie E. (2001). Revisiting the source of candidemia: Skin or gut?. Clin. Infect. Dis..

[7] Gouba N., Drancourt M. (2015). Digestive tract mycobiota: A source of infection. Med. Mal. Infect..

[8] Latge J.P., Chamilos G. (2019). *Aspergillus fumigatus* and Aspergillosis in 2019. Clin. Microbiol. Rev..

[9] Zhai B., Ola M., Rolling T., Tosini N.L., Joshowitz S., Littmann E.R., Amoretti L.A., Fontana E., Wright R.J., Miranda E. (2020). High-resolution mycobiota analysis reveals dynamic intestinal translocation preceding invasive candidiasis. Nat. Med..

[10] Pfaller M.A., Diekema D.J., Turnidge J.D., Castanheira M., Jones R.N. (2019). Twenty Years of the SENTRY Antifungal Surveillance Program: Results for *Candida* Species from 1997–2016. Open Forum Infect. Dis..

[11] Wisplinghoff H., Bischoff T., Tallent S.M., Seifert H., Wenzel R.P., Edmond M.B. (2004). Nosocomial bloodstream infections in US hospitals: Analysis of 24,179 cases from a prospective nationwide surveillance study. Clin. Infect. Dis..

[12] Xu S., Shinohara M.L. (2017). Tissue-Resident Macrophages in Fungal Infections. Front. Immunol..

[13] Qian Q., Jutila M.A., Van Rooijen N., Cutler J.E. (1994). Elimination of mouse splenic macrophages correlates with increased susceptibility to experimental disseminated candidiasis. J. Immunol..

[14] Sun D., Sun P., Li H., Zhang M., Liu G., Strickland A.B., Chen Y., Fu Y., Xu J., Yosri M. (2019). Fungal dissemination is limited by liver macrophage filtration of the blood. Nat. Commun..

[15] Leonardi I., Li X., Semon A., Li D., Doron I., Putzel G., Bar A., Prieto D., Rescigno M., McGovern D.P.B. (2018). CX3CR1(+) mononuclear phagocytes control immunity to intestinal fungi. Science.

[16] Kanayama M., Inoue M., Danzaki K., Hammer G., He Y.W., Shinohara M.L. (2015). Autophagy enhances NFkappaB activity in specific tissue macrophages by sequestering A20 to boost antifungal immunity. Nat. Commun..

[17] Lionakis M.S., Swamydas M., Fischer B.G., Plantinga T.S., Johnson M.D., Jaeger M., Green N.M., Masedunskas A., Weigert R., Mikelis C. (2013). CX3CR1-dependent renal macrophage survival promotes *Candida* control and host survival. J. Clin. Investig..

[18] Drummond R.A., Swamydas M., Oikonomou V., Zhai B., Dambuza I.M., Schaefer B.C., Bohrer A.C., Mayer-Barber K.D., Lira S.A., Iwakura Y. (2019). CARD9(+) microglia promote antifungal immunity via IL-1beta- and CXCL1-mediated neutrophil recruitment. Nat. Immunol..

[19] Moyes D.L., Wilson D., Richardson J.P., Mogavero S., Tang S.X., Wernecke J., Hofs S., Gratacap R.L., Robbins J., Runglall M. (2016). Candidalysin is a fungal peptide toxin critical for mucosal infection. Nature.

[20] Kleinegger C.L., Lockhart S.R., Vargas K., Soll D.R. (1996). Frequency, intensity, species, and strains of oral *Candida* vary as a function of host age. J. Clin. Microbiol..

[21] Soll D.R., Galask R., Schmid J., Hanna C., Mac K., Morrow B. (1991). Genetic dissimilarity of commensal strains of *Candida* spp. carried in different anatomical locations of the same healthy women. J. Clin. Microbiol..

[22] Ellepola A.N., Samaranayake L.P. (2001). Inhalational and topical steroids, and oral candidosis: A mini review. Oral Dis..

[23] Xu J., Schwartz K., Bartoces M., Monsur J., Severson R.K., Sobel J.D. (2008). Effect of antibiotics on vulvovaginal candidiasis: A MetroNet study. J. Am. Board Fam. Med..

[24] Hay R. (2018). Therapy of Skin, Hair and Nail Fungal Infections. J. Fungi.

[25] Wisplinghoff H., Ebbers J., Geurtz L., Stefanik D., Major Y., Edmond M.B., Wenzel R.P., Seifert H. (2014). Nosocomial bloodstream infections due to *Candida* spp. in the USA: Species distribution, clinical features and antifungal susceptibilities. Int. J. Antimicrob. Agents.

[26] Das I., Nightingale P., Patel M., Jumaa P. (2011). Epidemiology, clinical characteristics, and outcome of candidemia: Experience in a tertiary referral center in the UK. Int. J. Infect. Dis..

[27] Desai J.V., Lionakis M.S. (2018). The role of neutrophils in host defense against invasive fungal infections. Curr. Clin. Microbiol. Rep..

[28] Pappas P.G., Lionakis M.S., Arendrup M.C., Ostrosky-Zeichner L., Kullberg B.J. (2018). Invasive candidiasis. Nat. Rev. Dis. Prim..

[29] Archambault L.S., Trzilova D., Gonia S., Gale C., Wheeler R.T. (2019). Intravital Imaging Reveals Divergent Cytokine and Cellular Immune Responses to *Candida albicans* and *Candida parapsilosis*. mBio.

[30] Swidergall M., Khalaji M., Solis N.V., Moyes D.L., Drummond R.A., Hube B., Lionakis M.S., Murdoch C., Filler S.G., Naglik J.R. (2019). Candidalysin Is Required for Neutrophil Recruitment and Virulence During Systemic *Candida albicans* Infection. J. Infect. Dis..

[31] Drummond R.A., Collar A.L., Swamydas M., Rodriguez C.A., Lim J.K., Mendez L.M., Fink D.L., Hsu A.P., Zhai B., Karauzum H. (2015). CARD9-Dependent Neutrophil Recruitment Protects against Fungal Invasion of the Central Nervous System. PLoS Pathog..

[32] Heung L.J. (2020). Monocytes and the Host Response to Fungal Pathogens. Front. Cell Infect. Microbiol..

[33] Amon L., Lehmann C.H.K., Baranska A., Schoen J., Heger L., Dudziak D. (2019). Transcriptional control of dendritic cell development and functions. Int. Rev. Cell Mol. Biol..

[34] Gow N.A.R., Latge J.P., Munro C.A. (2017). The Fungal Cell Wall: Structure, Biosynthesis, and Function. Microbiol. Spectr..

[35] Gow N.A., Netea M.G., Munro C.A., Ferwerda G., Bates S., Mora-Montes H.M., Walker L., Jansen T., Jacobs L., Tsoni V. (2007). Immune recognition of *Candida albicans* beta-glucan by dectin-1. J. Infect. Dis..

[36] Walpole G.F.W., Grinstein S., Westman J. (2018). The role of lipids in host-pathogen interactions. IUBMB Life.

[37] Haas A. (2007). The phagosome: Compartment with a license to kill. Traffic.

[38] Wellington M., Dolan K., Krysan D.J. (2009). Live *Candida albicans* suppresses production of reactive oxygen species in phagocytes. Infect. Immun..

[39] Frohner I.E., Bourgeois C., Yatsyk K., Majer O., Kuchler K. (2009). *Candida albicans* cell surface superoxide dismutases degrade host-derived reactive oxygen species to escape innate immune surveillance. Mol. Microbiol..

[40] Dantas Ada S., Day A., Ikeh M., Kos I., Achan B., Quinn J. (2015). Oxidative stress responses in the human fungal pathogen, *Candida albicans*. Biomolecules.

[41] Lorenz M.C., Bender J.A., Fink G.R. (2004). Transcriptional response of *Candida albicans* upon internalization by macrophages. Eukaryot. Cell.

[42] Fradin C., De Groot P., MacCallum D., Schaller M., Klis F., Odds F.C., Hube B. (2005). Granulocytes govern the transcriptional response, morphology and proliferation of *Candida albicans* in human blood. Mol. Microbiol..

[43] Munoz J.F., Delorey T., Ford C.B., Li B.Y., Thompson D.A., Rao R.P., Cuomo C.A. (2019). Coordinated host-pathogen transcriptional dynamics revealed using sorted subpopulations and single macrophages infected with *Candida albicans*. Nat. Commun..

[44] Laurian R., Jacot-des-Combes C., Bastian F., Dementhon K., Cotton P. (2020). Carbon metabolism snapshot by ddPCR during the early step of *Candida albicans* phagocytosis by macrophages. Pathog. Dis..

[45] Westman J., Moran G., Mogavero S., Hube B., Grinstein S. (2018). *Candida albicans* Hyphal Expansion Causes Phagosomal Membrane Damage and Luminal Alkalinization. mBio.

[46] Vylkova S., Lorenz M.C. (2014). Modulation of phagosomal pH by *Candida albicans* promotes hyphal morphogenesis and requires Stp2p, a regulator of amino acid transport. PLoS Pathog..

[47] Rocha C.R., Schroppel K., Harcus D., Marcil A., Dignard D., Taylor B.N., Thomas D.Y., Whiteway M., Leberer E. (2001). Signaling through adenylyl cyclase is essential for hyphal growth and virulence in the pathogenic fungus *Candida albicans*. Mol. Biol. Cell.

[48] Silao F.G.S., Ward M., Ryman K., Wallstrom A., Brindefalk B., Udekwu K., Ljungdahl P.O. (2019). Mitochondrial proline catabolism activates Ras1/cAMP/PKA-induced filamentation in *Candida albicans*. PLoS Genet..

[49] Ghosh S., Navarathna D.H., Roberts D.D., Cooper J.T., Atkin A.L., Petro T.M., Nickerson K.W. (2009). Arginine-induced germ tube formation in *Candida albicans* is essential for escape from murine macrophage line RAW 264.7. Infect. Immun..

[50] Fernandez-Arenas E., Bleck C.K., Nombela C., Gil C., Griffiths G., Diez-Orejas R. (2009). *Candida albicans* actively modulates intracellular membrane trafficking in mouse macrophage phagosomes. Cell Microbiol..

[51] McKenzie C.G., Koser U., Lewis L.E., Bain J.M., Mora-Montes H.M., Barker R.N., Gow N.A., Erwig L.P. (2010). Contribution of *Candida albicans* cell wall components to recognition by and escape from murine macrophages. Infect. Immun..

[52] Wartenberg A., Linde J., Martin R., Schreiner M., Horn F., Jacobsen I.D., Jenull S., Wolf T., Kuchler K., Guthke R. (2014). Microevolution of *Candida albicans* in macrophages restores filamentation in a nonfilamentous mutant. PLoS Genet..

[53] Uwamahoro N., Verma-Gaur J., Shen H.H., Qu Y., Lewis R., Lu J., Bambery K., Masters S.L., Vince J.E., Naderer T. (2014). The pathogen *Candida albicans* hijacks pyroptosis for escape from macrophages. mBio.

[54] Wellington M., Koselny K., Sutterwala F.S., Krysan D.J. (2014). *Candida albicans* triggers NLRP3-mediated pyroptosis in macrophages. Eukaryot. Cell.

[55] Van de Veerdonk F.L., Joosten L.A., Netea M.G. (2015). The interplay between inflammasome activation and antifungal host defense. Immunol. Rev..

[56] Hise A.G., Tomalka J., Ganesan S., Patel K., Hall B.A., Brown G.D., Fitzgerald K.A. (2009). An essential role for the NLRP3 inflammasome in host defense against the human fungal pathogen *Candida albicans*. Cell Host Microbe.

[57] Tucey T.M., Verma J., Harrison P.F., Snelgrove S.L., Lo T.L., Scherer A.K., Barugahare A.A., Powell D.R., Wheeler R.T., Hickey M.J. (2018). Glucose Homeostasis Is Important for Immune Cell Viability during Candida Challenge and Host Survival of Systemic Fungal Infection. Cell Metab..

[58] Sudbery P., Gow N., Berman J. (2004). The distinct morphogenic states of *Candida albicans*. Trends Microbiol..

[59] Sudbery P.E. (2011). Growth of *Candida albicans* hyphae. Nat. Rev. Microbiol..

[60] Naglik J.R., Challacombe S.J., Hube B. (2003). *Candida albicans* secreted aspartyl proteinases in virulence and pathogenesis. Microbiol. Mol. Biol. Rev..

[61] Martchenko M., Alarco A.M., Harcus D., Whiteway M. (2004). Superoxide dismutases in *Candida albicans*: Transcriptional regulation and functional characterization of the hyphal-induced *SOD5* gene. Mol. Biol. Cell.

[62] Liu Y., Filler S.G. (2011). *Candida albicans* Als3, a multifunctional adhesin and invasin. Eukaryot. Cell.

[63] Richardson J.P., Ho J., Naglik J.R. (2018). *Candida*-Epithelial Interactions. J. Fungi.

[64] Moyes D.L., Richardson J.P., Naglik J.R. (2015). *Candida albicans*-epithelial interactions and pathogenicity mechanisms: Scratching the surface. Virulence.

[65] Allert S., Forster T.M., Svensson C.M., Richardson J.P., Pawlik T., Hebecker B., Rudolphi S., Juraschitz M., Schaller M., Blagojevic M. (2018). *Candida albicans*-Induced Epithelial Damage Mediates Translocation through Intestinal Barriers. mBio.

[66] Mitchell A.P. (2016). Microbiology: Fungus produces a toxic surprise. Nature.

[67] Birse C.E., Irwin M.Y., Fonzi W.A., Sypherd P.S. (1993). Cloning and characterization of ECE1, a gene expressed in association with cell elongation of the dimorphic pathogen *Candida albicans*. Infect. Immun..

[68] Martin R., Albrecht-Eckardt D., Brunke S., Hube B., Hunniger K., Kurzai O. (2013). A core filamentation response network in *Candida albicans* is restricted to eight genes. PLoS ONE.

[69] Bader O., Krauke Y., Hube B. (2008). Processing of predicted substrates of fungal Kex2 proteinases from *Candida albicans*, *C. glabrata*, *Saccharomyces cerevisiae* and *Pichia pastoris*. BMC Microbiol..

[70] Richardson J.P., Mogavero S., Moyes D.L., Blagojevic M., Kruger T., Verma A.H., Coleman B.M., De La Cruz Diaz J., Schulz D., Ponde N.O. (2018). Processing of *Candida albicans* Ece1p Is Critical for Candidalysin Maturation and Fungal Virulence. mBio.

[71] Gordon V.M., Leppla S.H. (1994). Proteolytic activation of bacterial toxins: Role of bacterial and host cell proteases. Infect. Immun..

[72] Gordon V.M., Klimpel K.R., Arora N., Henderson M.A., Leppla S.H. (1995). Proteolytic activation of bacterial toxins by eukaryotic cells is performed by furin and by additional cellular proteases. Infect. Immun..

[73] Abrami L., Fivaz M., Decroly E., Seidah N.G., Jean F., Thomas G., Leppla S.H., Buckley J.T., van der Goot F.G. (1998). The pore-forming toxin proaerolysin is activated by furin. J. Biol. Chem..

[74] Wilson D., Naglik J.R., Hube B. (2016). The Missing Link between *Candida albicans* Hyphal Morphogenesis and Host Cell Damage. PLoS Pathog..

[75] Gonzalez-Juarbe N., Gilley R.P., Hinojosa C.A., Bradley K.M., Kamei A., Gao G., Dube P.H., Bergman M.A., Orihuela C.J. (2015). Pore-Forming Toxins Induce Macrophage Necroptosis during Acute Bacterial Pneumonia. PLoS Pathog..

[76] Keyel P.A., Heid M.E., Salter R.D. (2011). Macrophage responses to bacterial toxins: A balance between activation and suppression. Immunol. Res..

[77] Cavaillon J.M. (2018). Exotoxins and endotoxins: Inducers of inflammatory cytokines. Toxicon.

[78] Kasper L., Konig A., Koenig P.A., Gresnigt M.S., Westman J., Drummond R.A., Lionakis M.S., Gross O., Ruland J., Naglik J.R. (2018). The fungal peptide toxin Candidalysin activates the NLRP3 inflammasome and causes cytolysis in mononuclear phagocytes. Nat. Commun..

[79] O’Meara T.R., Duah K., Guo C.X., Maxson M.E., Gaudet R.G., Koselny K., Wellington M., Powers M.E., MacAlpine J., O’Meara M.J. (2018). High-Throughput Screening Identifies Genes Required for *Candida albicans* Induction of Macrophage Pyroptosis. mBio.

[80] Krysan D.J., Sutterwala F.S., Wellington M. (2014). Catching fire: *Candida albicans,* macrophages, and pyroptosis. PLoS Pathog..

[81] Tucey T.M., Verma-Gaur J., Nguyen J., Hewitt V.L., Lo T.L., Shingu-Vazquez M., Robertson A.A., Hill J.R., Pettolino F.A., Beddoe T. (2016). The Endoplasmic Reticulum-Mitochondrion Tether ERMES Orchestrates Fungal Immune Evasion, Illuminating Inflammasome Responses to Hyphal Signals. mSphere.

[82] Gross O., Poeck H., Bscheider M., Dostert C., Hannesschlager N., Endres S., Hartmann G., Tardivel A., Schweighoffer E., Tybulewicz V. (2009). Syk kinase signalling couples to the Nlrp3 inflammasome for anti-fungal host defence. Nature.

[83] Joly S., Ma N., Sadler J.J., Soll D.R., Cassel S.L., Sutterwala F.S. (2009). Cutting edge: *Candida albicans* hyphae formation triggers activation of the Nlrp3 inflammasome. J. Immunol..

[84] Niemiec M.J., Grumaz C., Ermert D., Desel C., Shankar M., Lopes J.P., Mills I.G., Stevens P., Sohn K., Urban C.F. (2017). Dual transcriptome of the immediate neutrophil and *Candida albicans* interplay. BMC Genom..

[85] Tavares A.H., Burgel P.H., Bocca A.L. (2015). Turning Up the Heat: Inflammasome Activation by Fungal Pathogens. PLoS Pathog..

[86] Kelley N., Jeltema D., Duan Y., He Y. (2019). The NLRP3 Inflammasome: An Overview of Mechanisms of Activation and Regulation. Int. J. Mol. Sci..

[87] Gaidt M.M., Ebert T.S., Chauhan D., Schmidt T., Schmid-Burgk J.L., Rapino F., Robertson A.A., Cooper M.A., Graf T., Hornung V. (2016). Human Monocytes Engage an Alternative Inflammasome Pathway. Immunity.

[88] Van de Veerdonk F.L., Netea M.G., Dinarello C.A., Joosten L.A. (2011). Inflammasome activation and IL-1beta and IL-18 processing during infection. Trends Immunol..

[89] Place D.E., Muse S.J., Kirimanjeswara G.S., Harvill E.T. (2014). Caspase-1-independent interleukin-1β is required for clearance of *Bordetella pertussis* infections and whole-cell vaccine-mediated immunity. PLoS ONE.

[90] Joosten L.A., Netea M.G., Fantuzzi G., Koenders M.I., Helsen M.M., Sparrer H., Pham C.T., van der Meer J.W., Dinarello C.A., van den Berg W.B. (2009). Inflammatory arthritis in caspase 1 gene-deficient mice: Contribution of proteinase 3 to caspase 1-independent production of bioactive interleukin-1beta. Arthritis Rheum..

[91] Karmakar M., Sun Y., Hise A.G., Rietsch A., Pearlman E. (2012). Cutting edge: IL-1beta processing during *Pseudomonas aeruginosa* infection is mediated by neutrophil serine proteases and is independent of NLRC4 and caspase-1. J. Immunol..

[92] Mayer-Barber K.D., Barber D.L., Shenderov K., White S.D., Wilson M.S., Cheever A., Kugler D., Hieny S., Caspar P., Núñez G. (2010). Caspase-1 independent IL-1beta production is critical for host resistance to *Mycobacterium tuberculosis* and does not require TLR signaling in vivo. J. Immunol..

[93] Wellington M., Koselny K., Krysan D.J. (2012). *Candida albicans* morphogenesis is not required for macrophage interleukin 1beta production. mBio.

[94] Rogiers O., Frising U.C., Kucharikova S., Jabra-Rizk M.A., van Loo G., Van Dijck P., Wullaert A. (2019). Candidalysin Crucially Contributes to Nlrp3 Inflammasome Activation by *Candida albicans* Hyphae. mBio.

[95] Lowes D.J., Hevener K.E., Peters B.M. (2020). Second-Generation Antidiabetic Sulfonylureas Inhibit *Candida albicans* and Candidalysin-Mediated Activation of the NLRP3 Inflammasome. Antimicrob. Agents Chemother..

[96] Munoz-Planillo R., Kuffa P., Martinez-Colon G., Smith B.L., Rajendiran T.M., Nunez G. (2013). K(+) efflux is the common trigger of NLRP3 inflammasome activation by bacterial toxins and particulate matter. Immunity.

[97] Greaney A.J., Leppla S.H., Moayeri M. (2015). Bacterial Exotoxins and the Inflammasome. Front. Immunol..

[98] Craven R.R., Gao X., Allen I.C., Gris D., Bubeck Wardenburg J., McElvania-Tekippe E., Ting J.P., Duncan J.A. (2009). *Staphylococcus aureus* alpha-hemolysin activates the NLRP3-inflammasome in human and mouse monocytic cells. PLoS ONE.

[99] Cervantes J., Nagata T., Uchijima M., Shibata K., Koide Y. (2008). Intracytosolic *Listeria monocytogenes* induces cell death through caspase-1 activation in murine macrophages. Cell Microbiol..

[100] O’Meara T.R., Veri A.O., Ketela T., Jiang B., Roemer T., Cowen L.E. (2015). Global analysis of fungal morphology exposes mechanisms of host cell escape. Nat. Commun..

[101] Pietrella D., Pandey N., Gabrielli E., Pericolini E., Perito S., Kasper L., Bistoni F., Cassone A., Hube B., Vecchiarelli A. (2013). Secreted aspartic proteases of *Candida albicans* activate the NLRP3 inflammasome. Eur. J. Immunol..

[102] Richardson J.P., Willems H.M.E., Moyes D.L., Shoaie S., Barker K.S., Tan S.L., Palmer G.E., Hube B., Naglik J.R., Peters B.M. (2018). Candidalysin Drives Epithelial Signaling, Neutrophil Recruitment, and Immunopathology at the Vaginal Mucosa. Infect. Immun..

[103] Casadevall A., Pirofski L.A. (2014). Microbiology: Ditch the term pathogen. Nature.

[104] Pellon A., Sadeghi Nasab S.D., Moyes D.L. (2020). New Insights in *Candida albicans* Innate Immunity at the Mucosa: Toxins, Epithelium, Metabolism, and Beyond. Front. Cell Infect. Microbiol..

[105] Naglik J.R., Moyes D.L., Wachtler B., Hube B. (2011). *Candida albicans* interactions with epithelial cells and mucosal immunity. Microbes Infect..

[106] Verma A.H., Richardson J.P., Zhou C., Coleman B.M., Moyes D.L., Ho J., Huppler A.R., Ramani K., McGeachy M.J., Mufazalov I.A. (2017). Oral epithelial cells orchestrate innate type 17 responses to *Candida albicans* through the virulence factor candidalysin. Sci. Immunol..

[107] Ho J., Yang X., Nikou S.A., Kichik N., Donkin A., Ponde N.O., Richardson J.P., Gratacap R.L., Archambault L.S., Zwirner C.P. (2019). Candidalysin activates innate epithelial immune responses via epidermal growth factor receptor. Nat. Commun..

[108] Ho J., Wickramasinghe D.N., Nikou S.A., Hube B., Richardson J.P., Naglik J.R. (2020). Candidalysin Is a Potent Trigger of Alarmin and Antimicrobial Peptide Release in Epithelial Cells. Cells.

[109] Siscar-Lewin S., Hube B., Brunke S. (2019). Antivirulence and avirulence genes in human pathogenic fungi. Virulence.

[110] Naglik J.R., Gaffen S.L., Hube B. (2019). Candidalysin: Discovery and function in *Candida albicans* infections. Curr. Opin. Microbiol..

[111] Lionakis M.S., Lim J.K., Lee C.C., Murphy P.M. (2011). Organ-specific innate immune responses in a mouse model of invasive candidiasis. J. Innate Immun..

[112] Romo J.A., Kumamoto C.A. (2020). On Commensalism of *Candida*. J. Fungi.

[113] Witchley J.N., Penumetcha P., Abon N.V., Woolford C.A., Mitchell A.P., Noble S.M. (2019). *Candida albicans* Morphogenesis Programs Control the Balance between Gut Commensalism and Invasive Infection. Cell Host Microbe.

[114] Ruben S., Garbe E., Mogavero S., Albrecht-Eckardt D., Hellwig D., Hader A., Kruger T., Gerth K., Jacobsen I.D., Elshafee O. (2020). Ahr1 and Tup1 Contribute to the Transcriptional Control of Virulence-Associated Genes in *Candida albicans*. mBio.

[115] Romo J.A., Zhang H., Cai H., Kadosh D., Koehler J.R., Saville S.P., Wang Y., Lopez-Ribot J.L. (2019). Global Transcriptomic Analysis of the *Candida albicans* Response to Treatment with a Novel Inhibitor of Filamentation. mSphere.

